# Employment and financial burden of families with preschool children diagnosed with autism spectrum disorders in urban China: results from a descriptive study

**DOI:** 10.1186/s12888-015-0382-4

**Published:** 2015-01-22

**Authors:** Jian-Jun Ou, Li-Juan Shi, Guang-Lei Xun, Chen Chen, Ren-Rong Wu, Xue-Rong Luo, Feng-Yu Zhang, Jing-Ping Zhao

**Affiliations:** Mental Health Institute of The Second Xiangya Hospital and Key Laboratory of Psychiatry and Mental Health of Hunan Province, The Central South University, 139 Middle Renmin Road, Changsha, 410011 Hunan P.R. China; Shandong Mental Health Center, 49 East Wenhua Road, Jinan, 250014 Shandong P.R. China; Shanghai Key Laboratory of Forensic Medicine, Institute of Forensic Science, The Ministry of Justice, 1347 West Guang Fu Road, Shanghai, 200063 P.R. China; Division of Clinical Sciences, Lieber Institute for Brain Development, John Hopkins University Medical Campus, 855 N. Wolfe Street, Suite 300, Baltimore, MD 21205 USA

**Keywords:** Employment burden, Financial burden, Autism spectrum disorder, Chinese

## Abstract

**Background:**

Autism spectrum disorder (ASD) affects many aspects of family life, such as social and economic burden. Little investigation of this phenomenon has been carried out in China. We designed this study to evaluate the employment and financial burdens of families with ASD-diagnosed preschoolers.

**Methods:**

Four hundred and fifty-nine nuclear families of children with ASD, 418 with some other disability (OD) and 424 with typically developing (TD) children were recruited for this study. Employment and financial burdens of families were evaluated using a structured questionnaire; logistic regression was used to examine differences in job change measures by group, and ordinal logistic regression was used to investigate the association between household income and group.

**Results:**

Fifty-eight percent of families with ASD children and 19% of families with OD children reported that childcare problems had greatly affected their employment decisions, compared with 9% of families with TD children (p < 0.001). Age of child, parental education and parental age notwithstanding, having a child with ASD and having a child with OD were both associated with increased odds of reporting that childcare greatly interfered with employment (ASD, OR: 15.936; OD, OR: 2.502; all p < 0.001) and decreased the odds of living in a higher-income household (ASD, estimate = -1.271; OD, estimate = -0.569; all p < 0.001). The average loss of annual income associated with having a child with ASD was Chinese RenMinBi (RMB) 44,077 ($7,226), compared with RMB 20,788 ($3,408) for families of OD children.

**Conclusions:**

ASD is associated with severe employment and financial burdens, much more than for OD, in families with preschool children.

## Background

Autism spectrum disorder (ASD) is a pervasive developmental disorder characterized by persistent deficits in social communication and social interaction, and manifested restricted, repetitive patterns of behavior, interests, or activities [1]. This disease presents in the early life stage of 2–6 years, and its prevalence has increased rapidly over past decades, from a median global prevalence of about 4.5 per 10,000 [2] to 62 per 10,000 [3], with some estimates even higher, at 120 to 260 per 10,000 [4-7]. Although this rapid increase in prevalence may be partly due to more frequent diagnosis and increased awareness of autism [8], the latest evidence from the United States shows that the prevalence of ASD was 2.00% in 2011–2012, a significant increase from 1.16% in 2007 [9]. This supports the idea that the increase represents an actual change in the prevalence of autism. ASD has become one of the most common childhood disorders or health conditions.

ASD affects many aspects of family life, not only leading to physical and psychological impairment of parents [10-12], but also causing great unemployment and financial burdens. In previous studies, 39% of parents of children with ASDs have reported that childcare problems greatly affected their employment decisions, and that the average loss of annual income associated with having a child with ASDs was $6,200 or 14% of their reported income in the United States [13,14]. A recent study also found that children with ASD are 9% less likely to have both parents employed in the United States, and that family earnings are 21% ($10,416) less than those of families with children who have another health limitation and 28% ($17,763) less than those of families with children who have no health limitation [15]. When compared with other childhood diseases, ASD was more likely to be associated with financial problems, a need for additional income to provide for the child’s medical care, a reduction or cessation of work because of the child’s condition, an investment of 10 or more hours per week providing or coordinating care, and payments of more than $1000 in the previous year for the child’s care [16]. Furthermore, the high cost of ASD-specific education may make the financial situation of ASD families worse, and some low-income families are unable to afford it at all [17,18].

In many employment and financial burden-related factors, ASD emerges as the most important influence. Montes et al. [13] controlled for household and child covariates and found that families with a child with ASD were seven times more likely than other families to state that childcare problems affected employment (OR: 7.31; 95% CI: 3.44–15.54). This effect was three times larger than that of poverty (OR: 2.02; 1.57–2.59). In another study [14], having a child with ASD (OR: 0.62; 95% CI: 0.43–0.91) significantly decreased the odds of living in a higher-income household, even after variables of parental education (OR: 2.28; 95% CI: 2.17–2.40), type of family (two-parent family, OR: 3.71; 95% CI: 3.24–4.25), parental age, location of the household (urban, OR: 1.44; 95% CI: 1.26–1.65), and minority ethnicity (child is white, non-Hispanic, OR: 1.99; 95% CI: 1.76–2.23) were controlled for.

While family care studies of ASD patients have been carried out in many countries, few data have been collected in China [19]. Although autism has recently drawn special increased awareness in China [20,21], we still do not know how severely autistic patients affect their families’ employment and finances, or what specific factors may be associated with these burdens. Therefore, we designed this descriptive study to evaluate parental employment and financial burdens of the family and to examine related factors that may be linked to these employment and financial burdens. This study aims not only to provide information that may be used for increasing public awareness of ASD, but also to provide some empirical data to help policy makers prioritize health resources for effective intervention and education on autism in China.

## Methods

### Subjects

Nuclear families with children diagnosed with ASD were recruited at three large public/private special education schools if their children met the following criteria: (1) age 3–6 years and has been diagnosed with ASD in a local hospital; (2) diagnosis of ASD was made using the Diagnostic and Statistical Manual of Mental Disorders, Fourth Edition, Text Revision (DSM-IV-TR), and was confirmed by a senior child psychiatrist through a face-to-face observation and interview with parents. To reduce possible bias in estimating costs that may be due to other diseases, families with children that had severe physical diseases such as congenital heart disease or hematological system diseases were excluded. Chromosome morphology through karyotype analysis was performed to exclude some common chromosomal diseases such as Down’s syndrome and Chromosome 18 duplication.

Nuclear families with children diagnosed with some other disability (OD) were recruited from special education schools, and nuclear families with typically developing (TD) children were recruited from ordinary kindergartens as two comparison groups. The inclusion criteria for the OD group were as follows: (1) age 3–6 years; (2) child did not have ASD and did have any of the following conditions: a specific learning or language disability, mental retardation, serious emotional disturbance, serious hearing or visual impairment, an orthopedic impairment, attention-deficit disorder or attention-deficit/hyperactivity disorder and other health impairment lasting 6 months or more; (3) diagnosis of OD was confirmed using proof of diagnosis from hospital. Inclusion criteria for the TD group were as follows: (1) age 3–6 years; (2) performing well in kindergarten and without any parents’ or teachers’ reports of serious emotional or behavioral problems.

Families with children who had severe physical diseases such as congenital heart disease or hematological system diseases were excluded from the study, as were families whose parents had either severe physical or mental diseases that may seriously affect their ability to work.

Families with ASD children were recruited from two special education schools in Hunan province and one in Shandong province. Because these schools recruit students from all over the country, recruited families with ASD children came from 27 provinces (but mainly from Hunan and Shandong province). While Shandong is a high-income province, Hunan is a middle-income province, and some other provinces are low-income provinces. Comparison groups were recruited from another high-income province (Jiangsu), a middle-income province (Hunan), and some low-income provinces such as Guangxi, Henan and Yunnan. To reduce the geographic difference in macroeconomic development, we calculated the weighted average of annual household income for provinces from which ASD families were recruited, then selected comparison samples to match these cases.

The study was approved by the ethics committee of the Second Xiangya Hospital, and conducted between January 2011 and December 2012. All families provided written informed consent before enrolment.

### Measures

Outcome measures were employment burden of parents with ASD children and financial burden of the families. Both measures were assessed with a structured questionnaire. Because the socioeconomic status of parents, family medical history and demographic information were related to employment and financial burdens, this information was also collected. Employment burden was measured by a single item that asked whether the father or mother suffered job loss or job change mainly because of a childcare problem in the past 12 months. A binary variable was coded as 0 if the job was not affected, and 1 if job loss or change had occurred.

Financial burden was measured in the context of family income. An open question was asked to estimate household income in the past year: “How much total income did all family members earn in the past year, including salaries, non-salary and other earnings, interest revenues, pensions, and so on?” To verify the accuracy of household income prediction model, an additional open question was asked of families with children diagnosed with ASD or OD: “How much total income do you expect that all family members would have earned in the past year if your child did not have the disease? (You can use the household income of your colleague-peers who have the same level of education as a reference)”.

In addition, educational expenses were collected to measure the direct cost of children. An open question asked: “What were the total educational expenses of the ASD or OD child in your household over the past year, including general/special school education, extracurricular practice, related books/materials, and so on?”

Basic sociodemographic and family medical history information was collected for all family members to control for possible confounding. These general family characteristics included family demographics and structure, medical history, housing conditions, history of pregnancy and child development. The level of parental education was also collected to measure the socioeconomic status in the family; this variable was classified into three categories: below high school, high school or equivalent, and above high school.

### Statistical analysis

Because employment status was coded as a binary outcome, cross-tabulation with the Chi-Square test and logistic regression were used to examine the association of disease group with the change in employment status while controlling for parental socioeconomic status factors such as education and demographics.

In China, different areas have different regional development levels, and these regional differences affect household income directly. Therefore, analyzing financial burden, we took some steps to match the comparison samples with the autistic sample on regional differences in macroeconomic status. We first calculated the weighted average of annual household income based on the economic data by province in 2011 and 2012 according to the National Bureau of Statistics of China [22]. Annual household income was calculated according to the average number of employees in a family, wage income per employee and the proportion of total revenue accounted for by wage income. The number of ASD families in each province was used as a weight. ASD families from extremely high- or low-income provinces were then removed, and comparison samples from certain provinces with similar average annual household incomes were selected for analysis.

To calculate the loss of income in families with ASD or OD children, we built a linear regression model using a sample of families with TD children to obtain the predicted household income in the ASD and OD groups based on parental socioeconomic status and demographics. The predicted household income of families with ASD or OD children was calculated with the linear regression model. Average loss of income was then calculated by taking average predicted household income minus average actual household income.

Ordinal logistic regression was used to examine the association of the reported actual household income with groups, while the age of the child and parental age and education were controlled as covariates. In this process, annual household income was converted into an ordinal variable: 1 if less than ¥30,000 ($4,917), which denotes extremely low income; 2 if ¥30,000 to 49,999 ($4,918 to 8,196), which denotes low income; 3 if ¥50,000 to 69,999 ($8,197 to 11,475), which denotes moderate income; 4 if ¥70,000 to 99,999 ($11,476 to 16,393), which denotes high income; and 5 if more than ¥100,000 ($16,394), which denotes extremely high income.

Income was described as a mean, with the local currency unit (Chinese Yuan, ¥) and the international common currency unit (U.S. dollars, $). An exchange rate of ¥6.1 = $1.0 was used. All statistical tests were two-tailed, with 0.05 as the level of significance. SPSS 19.0 for Windows was used for statistical analysis.

## Results

After eligibility screening, 459 of 493 (93%) ASD families, 418 of 492 (85%) OD families, and 424 of 470 (90%) TD families were enrolled. There were no significant differences in the general characteristics of children or their parents, except gender of children, across the three groups (Table 1).

**Table 1:** **Comparison of general information among three groups** ^*^

	**Children with ASD (N = 459)**		**Children with other disability (N = 418)**		**Typically developing children (N = 424)**		**F(2, 1300)/** ***χ*** ^**2**^	**p**
	**Mean or N**	**SD or %**	**Mean or N**	**SD or %**	**Mean or N**	**SD or %**		
Age of children, years, Mean (SD)	4.32	1.085	4.31	0.998	4.29	0.973	0.118	0.889
Composition of children								
Boy, N (%)	399	86.9	295	70.6	228	53.8	117.377	<0.001
Girl, N (%)	60	13.1	123	29.4	196	46.2		
Age of parents, years, Mean (SD)								
Father	34.15	4.04	34.64	4.16	34.36	4.85	1.367	0.255
Mother	32.37	3.44	32.34	3.87	31.88	4.37	2.114	0.121
Education of father, N (%)								
Lower than high school	56	12.2	66	15.8	74	17.5	7.013	0.135
High school or equivalent	74	16.1	79	18.9	69	16.3		
Higher than high school	329	71.7	273	65.3	281	66.3		
Education of mother, N (%)								
Lower than high school	70	15.3	76	18.2	89	21.0	5.623	0.229
High school or equivalent	103	22.4	82	19.6	88	20.8		
Higher than high school	286	62.3	260	62.2	247	58.3		

Note: SD, standard deviation; *Continuous data were analyzed by ANOVA, and qualitative data were analyzed by *χ* ^2^ test.

**Figure 1:**
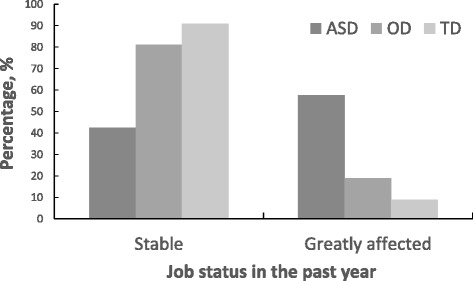
**The comparison of job status in the past year among the three groups.** Significant group differences were observed, *χ* ^2^ = 282.984, p < 0.001.

The employment burden was significantly different across ASD, OD and TD families. For ASD families, 57.5% (264/459) reported that childcare problems had affected their jobs in the past year. Of those, 240 families (52.3%) reported that someone had quit or turned down a job, and 24 (5.2%) reported that someone had changed jobs. Meanwhile, 79 of 418 (18.9%) OD families reported job effects, of which 70 (16.7%) were jobs lost or not taken, and nine (2.2%) were job changes. In contrast, in the family with TD children, only a small number of families (38/424 = 8.9%) reported that childcare problems affected their jobs; of these, 26 (6.1%) were jobs lost or not taken, and 12 (2.8%) were job changes. Differences in the employment burden among the three groups were significant (*χ*^2^ = 282.984, p < 0.001) (Figure 1).

After controlling for sociodemographic variables, we still found that ASD families were more likely to report that childcare affected their employment status. Compared with families with TD children, ASD families experienced a 15-fold increase in the likelihood of job problems in the past 12 months (OR: 15.94, 95% CI: 10.73–23.66, p < 0.001), whereas having a child with OD was associated with a far lower, but still significantly elevated, likelihood of job effects (OR: 2.502, 95% CI: 1.643–3.809, p < 0.001). A significant difference in job effects was also observed between ASD and OD groups (Wald test F = 129.830, p < 0.001). We also noted that a higher education level of fathers was negatively associated with employment burden. Families with fathers who had high school or equivalent (OR: 0.594, 95% CI: 0.358–0.985) or above high school education (OR: 0.507, 95% CI: 0.297–0.863) were less likely than those with lower education levels to have employment effects (Table 2).

**Table 2:** **Logistic regression analysis for the impact of employment**

**Variable**	**Estimate**	**SE**	**p value**	**OR**	**95% CI for OR**		
					**Lower**	**Upper**	
Age							
Child	0.023	0.068	0.733	1.023	0.896	1.168	
Father	-0.016	0.017	0.341	0.984	0.953	1.017	
Mother	<0.001	0.019	0.998	1.000	0.963	1.038	
Education of father							
Below high school (reference)							
High school or equivalent	-0.521	0.258	0.043	0.594	0.358	0.985	*
Higher than high school	-0.680	0.272	0.012	0.507	0.297	0.863	*
Education of mother							
Below high school (reference)							
High school or equivalent	0.107	0.248	0.667	1.113	0.684	1.811	
Higher than high school	-0.430	0.267	0.107	0.650	0.385	1.098	
Disease group							
Typical development (reference)							
ASD	2.769	0.202	<0.001	15.936	10.734	23.658	**
Other disability	0.917	0.214	<0.001	2.502	1.643	3.809	**

ASD and OD groups are significantly different (Wald test F = 129.830, p < 0.001).

*p < 0.05, **p < 0.001.

In analyzing financial burden, we found that the annual household incomes of Beijing and Shanghai were much higher than in other provinces, so ASD patients from Beijing and Shanghai were excluded from the analysis. The weighted average of annual household income in ASD patients was ¥95,509 ($15,657), very close to that of ¥95,459 ($15,649) in Hunan province. Therefore, we selected the samples of Hunan province as comparisons of financial burden. The final analysis included 449 families in the ASD group, 348 families in the OD group and 281 families in the TD group. There were no significant group differences in the general characteristics of children and their parents, except the proportion of gender in children (*χ*^2^ = 109.174, p < 0.001) and education of the mother (*χ*^2^ = 12.173, p = 0.016).

The linear regression model provided reasonable predictions of average annual household income of the ASD and OD group. Through stepwise regression of TD samples, only the age of the child and education of parents entered the regression model using a criteria of probability of F entry = 0.05 and removal = 0.10. Based on this model, the mean predicted annual household income of all samples (TD, ASD and OD) was ¥107,375 ($17,603), very close to the mean reported annual household income (¥103,230, $16,923) of urban Chinese families from 2011 (¥97,362, $15,961) to 2012 (¥109,097, $17,885) (Table 3). The difference of ¥4,145 ($680) was within 5% of the reported annual income of all samples. Furthermore, the mean predicted annual income by model was ¥105,638 ($17,318) for the ASD group and ¥109,828 ($18,005) for the OD group, which were also very close to the mean reported expected household annual income of both ASD (¥102,799, $16,852) and OD (¥107,845, $17,680) (Table 3). The difference was also within 5% of the expected annual household income of each sample.

**Table 3:** **Estimated loss of income associated with having a child with ASD or OD**

	**Annual income (2011-2012)**			
	**All families (TD, ASD and OD)**	**Families with TD child**	**Families with ASD child**	**Families with OD child**
Predicted based on model	¥107,375 ($17,603)	¥107,112 ($17,559)	¥105,638 ($17,318)	¥109,828 ($18,005)
Reported expected income	-	-	¥102,799 ($16,852)	¥107,845 ($17,680)
Reported actual income	¥103,230, ($16,923)^a^	¥107,112 ($17,559)	¥61,561 ($10,092)	¥89,040 ($14,597)
Loss of income (difference between predicted and reported)	¥4,145 ($680)	-	¥44,077 ($7,226)	¥20,788 ($3,408)

^a^The mean annual income of Chinese urban families from 2011 (¥97,362, $15,961) to 2012 (¥109,097, $17,885). Data are from the National Bureau of Statistics of China.

The reported mean annual income was only ¥61,561 ($10,092) for families with ASD children and ¥89,040 ($14,597) for families with OD children, both significantly less than the reported annual income of ¥107,112 ($17,559) in families with TD children (post hoc analysis, all p < 0.005). The difference of ¥44,077 ($7,226) between the reported and predicted income constitutes a 41.7% loss of income associated with ASD. In contrast, the difference of ¥20,788 ($3408) between the reported and predicted income only constitutes an 18.9% loss of income associated with OD (Table 3).

The household income was decreased in families with ASD and OD children. Ordinal logistic regression showed that compared with families with TD children, both ASD and OD families reported significant decreases in their reported actual household income after controlling for the age of the child and parental age and education (ASD, estimate = -1.271, p < 0.001; OD, estimate = -0.569, p < 0.001). There was also a significant difference in the reported household incomes between the ASD and OD groups (Wald test F = 26.515; p < 0.001). We also noted that the age of the child was negatively associated with household income, whereas parental education was positively associated with household income (Table 4).

**Table 4:** **Ordinal logistic regression analysis for reported actual household income**

**Variable**	**Estimate**	**SE**	**p value**	**95% CI for estimate**		
				**Lower**	**Upper**	
Age						
Child	-0.141	0.054	0.009	-0.247	-0.036	*
Father	0.030	0.014	0.031	0.003	0.057	*
Mother	0.009	0.015	0.544	-0.020	0.037	
Education of father						
Below high school (reference)						
High school or equivalent	1.068	0.252	<0.001	0.574	1.562	**
Higher than high school	1.645	0.260	<0.001	1.136	2.154	**
Education of mother						
Below high school (reference)						
High school or equivalent	0.486	0.237	0.040	0.022	0.951	*
Higher than high school	1.196	0.244	<0.001	0.717	1.675	**
Disease group						
Typical development (reference)						
ASD	-1.271	0.144	<0.001	-1.553	-0.988	**
Other Disability	-0.596	0.149	<0.001	-0.888	-0.304	**

ASD and OD groups are significantly different (Wald test F = 26.515; p < 0.001).

*p < 0.05, **p < 0.001.

The cost of education was another important aspect of financial burden. The mean annual cost of education for children was ¥55,359 ($9,075) or 89.9% of the reported annual income in families with ASD children. This was significantly higher than that reported by both OD families, ¥27,904 ($4,574) or 31.3% of reported annual income (p < 0.001), and TD families, ¥17,413 ($2,855) or 16.3% of reported annual income (p < 0.001). Furthermore, the number of families with negative net income (after education expenses) was 199 (44.3%) in ASD families, just seven (2.0%) in OD families and one (0.4%) in TD families (Fisher exact test, value = 350.938, p < 0.001).

## Discussion

In this study, we used two comparison groups to evaluate the employment and financial burdens of families with preschool children diagnosed with ASD in urban China. We found that families with an ASD child are more likely than families with an OD or TD child to report employment effects due to childcare problems. Families with an ASD child were also more likely to be in substantially lower-income households, and to have costly educational expenses.

Although we did not actually conduct formal comparisons between our results and those of other studies, some informal comparisons can be made. The childcare burden of ASD families was heavier and more likely to disrupt parental employment in China than in the West. In our study, more than half of families with ASD children reported that their employment had been disrupted by autism-related childcare difficulties in the past year; this burden was 16-fold greater than for TD families. This number was higher than the reported 39% of job effects and seven-fold difference in burden reported in the United States [13]. The differences may be due to inadequate healthcare services for ASD families in China. The heavy care burden for ASD children leads to families needing additional support services [16]. Unfortunately, these kinds of services are very limited in China—more limited, in fact, than for other disabilities. Caregivers, typically mothers, have to be with their autistic children by themselves every moment in daily life, even when the child attends teaching in special education schools. This model is very common, and is more likely to bring serious disruptions in employment conditions for many families. In contrast, we found that the employment burden for families with OD children is only slightly greater than for families with TD children, but significantly less than for families with ASD children. Culture-related childcare (grandparents to help look after the children), a lighter care burden from OD, and an adequate support services system may have contributed to the smaller effect.

We expect that the heavy employment burden of ASD families affected financial burden strongly and directly. Our results suggest that families with a child with ASD are more likely to have lower income than are families with OD or TD children, and loss of employment is a very important driver of low income. These findings are consistent with previous studies, which imply that loss of income may stem from poorer-than-expected labor market performance, lower-than-expected labor participation, and lower-than-expected savings and investments [14]. We also found some other factors associated with household income. Consistent with many previous studies, our results showed that parents’ higher education was significantly associated with higher income. We speculate that this finding could well explain the negative correlation between paternal education and employment burden. Families with high income could hire someone to look after their children with ASD or OD, whereas families with low income may be more likely to give up their jobs in favor of caring for their children. The significant negative correlation between the age of the child and income of families may indirectly reflect the effect of care problems, which increased with age. The effect of paternal age was very limited in the model, and we speculate that it may be associated with a long working life. Usually the longer working life, the higher the income.

Families with ASD children face a substantial financial burden, which also stems from the high cost of education. Although early school training is very important for the development of children with autism [23], it is also very expensive. In our study, the mean annual cost of school training for children with ASD was significantly higher than that for TD or OD children. Moreover, more than 40% of families with ASD children had negative net income after education expenses, which means that these families cannot afford such education for very long. These findings were consistent with a previous study in the United States [24]. Unfortunately, the unfavorable financial situation may be more severe than our findings suggest, because some previous studies have demonstrated that families of children with ASD also have substantial healthcare expenses [25,26]. Therefore, more financial and policy supports are necessary for families with ASD children.

### Study limitations

The first limitation is that families with children diagnosed with ASD were recruited from special education schools; therefore low-income families, who cannot afford the cost of such schools, were not represented. The second limitation is that our study only investigated the very short-term employment and financial burdens of families with children diagnosed with ASD, so changes in such burdens over time could not be evaluated. Third, this study mainly investigated indirect economic burdens. The addition of surveys on direct economic burdens, such as medical expenditures, would help us understand the economic burdens of ASD families more comprehensively. Therefore, low-income families with ASD children should also be recruited, studies should investigate long-term effects, and more surveys about direct economic burdens should be conducted.

## Conclusions

ASD is associated with severe employment and financial burdens, much more than OD, in Chinese families with preschool children. More support services and resources should be provided to these families.

## References

[1] American Psychiatric Association (2013). Diagnostic and statistical manual of mental disorders.

[2] Lotter V (1966). Epidemiology of autistic conditions in young children. Soc Psychiatry.

[3] Elsabbagh M, Divan G, Koh YJ, Kim YS, Kauchali S, Marcin C (2012). Global prevalence of autism and other pervasive developmental disorders. Autism Res.

[4] Saemundsen E, Magnusson P, Georgsdottir I, Egilsson E, Rafnsson V (2013). Prevalence of autism spectrum disorders in an Icelandic birth cohort. BMJ Open.

[5] Russell G, Rodgers LR, Ukoumunne OC, Ford T (2013). Prevalence of parent-reported ASD and ADHD in the UK: findings from the millennium cohort study. J Autism Dev Disord.

[6] Autism and Developmental Disabilities Monitoring Network Surveillance Year 2008 Principal Investigators, Centers for Disease Control and Prevention (2012). Prevalence of autism spectrum disorders--autism and developmental disabilities monitoring network, 14 sites, United States, 2008. Morb Mortal Wkly Rep Surveill Summ.

[7] Kim YS, Leventhal BL, Koh YJ, Fombonne E, Laska E, Lim EC (2011). Prevalence of autism spectrum disorders in a total population sample. Am J Psychiatry.

[8] Fombonne E (2009). Epidemiology of pervasive developmental disorders. Pediatr Res.

[9] Blumberg SJ, Bramlett MD, Kogan MD, Schieve LA, Jones JR, Lu MC (2013). Changes in prevalence of parent-reported autism spectrum disorder in school-aged US children: 2007 to 2011–2012. Natl Health Stat Rep.

[10] Schieve LA, Blumberg SJ, Rice C, Visser SN, Boyle C (2007). The relationship between autism and parenting stress. Pediatrics.

[11] Mugno D, Ruta L, D'Arrigo VG, Mazzone L (2007). Impairment of quality of life in parents of children and adolescents with pervasive developmental disorder. Health Qual Life Outcomes.

[12] Stuart M, McGrew JH (2009). Caregiver burden after receiving a diagnosis of an autism spectrum disorder. Autism Res.

[13] Montes G, Halterman JS (2008). Childcare problems and employment among families with preschool-aged children with autism in the United States. Pediatrics.

[14] Montes G, Halterman JS (2008). Association of childhood autism spectrum disorders and loss of family income. Pediatrics.

[15] Cidav Z, Marcus SC, Mandell DS (2012). Implications of childhood autism for parental employment and earnings. Pediatrics.

[16] Kogan MD, Strickland BB, Blumberg SJ, Singh GK, Perrin JM, van Dyck PC (2008). A national profile of the health care experiences and family impact of autism spectrum disorder among children in the United States, 2005-2006. Pediatrics.

[17] Peters-Scheffer N, Didden R, Korzilius H, Matson J (2012). Cost comparison of early intensive behavioral intervention and treatment as usual for children with autism spectrum disorder in the Netherlands. Res Dev Disabil.

[18] Barrett B, Byford S, Sharac J, Hudry K, Leadbitter K, Temple K (2012). Service and wider societal costs of very young children with autism in the UK. J Autism Dev Disord.

[19] Wang J, Zhou X, Xia W, Sun CH, Wu LJ, Wang JL (2012). Parent-reported health care expenditures associated with autism spectrum disorders in Heilongjiang province, China. BMC Health Serv Res.

[20] Wang J, Hu Y, Wang Y, Qin X, Xia W, Sun C (2013). Parenting stress in Chinese mothers of children with autism spectrum disorders. Soc Psychiatry Psychiatr Epidemiol.

[21] Xia K, Guo H, Hu Z, Xun G, Zuo L, Peng Y (2013). Common genetic variants on 1p13.2 associate with risk of autism. Mol Psychiatry.

[22] National Bureau of Statistics of China (2013). China statistical yearbook 2012.

[23] Warren Z, McPheeters ML, Sathe N, Foss-Feig JH, Glasser A, Veenstra-Vanderweele J (2011). A systematic review of early intensive intervention for autism spectrum disorders. Pediatrics.

[24] Chambers JG, Kidron Y, Spain AK (2004). Characteristics of high-expenditure students with disabilities, 1999-2000. Report 8.

[25] Croen LA, Najjar DV, Ray GT, Lotspeich L, Bernal P (2006). A comparison of health care utilization and costs of children with and without autism spectrum disorders in a large group-model health plan. Pediatrics.

[26] Liptak GS, Stuart T, Auinger P (2006). Health care utilization and expenditures for children with autism: data from U.S. national samples. J Autism Dev Disord.

